# Communication skill training in surgical residency: insights from *Y-SICO* (Young-Italian Society of Surgical Oncology)

**DOI:** 10.1007/s13304-026-02557-2

**Published:** 2026-04-10

**Authors:** Rossella Melcarne, Federico Cappellacci, Filippo Carannante, Ludovico Carbone, Federica Ferracci, Stefano Fracon, Michele Manigrasso, Erica Milone, Edoardo Maria Muttillo, Stefania Piccioni, Silvia Sofia, Giulia Turri, Marcello Deraco, Antonio Macrì, Franco Roviello, COSTRUIRE-Collaborative Group, Caterina Accardo, Elisa Agus, Luca Alberti, Maria Ida Amabile, Carla Ammendolia, Giorgio Ammerata, Paolo Amoretti, Felicia Andrei, Michela Angelucci, Alfredo Annicchiarico, Pietro Anoldo, Laura Antolino, Valerio Argiolas, Mirko Armas, Enrica Avezzù, Andrea Baldo, Lorenzo Barberis, Maria Rachele Barbieri, Giulia Becherucci, Elisa Bertilone, Chiara Bettini, Massimo Biondo, Francesca Blasa, Andrea Boniotti, Giacomo Borroni, Alessandra Bozza, Greta Bracchetti, Francesco Brucchi, Angela Bucaro, Anna Bella Burciu, Fabiana Caciolo, Riccardo Calef, Matteo Calì, Gaetano Silvio Calleri, Federico Cammillini, Vincenzo Canalella, Fabio Carbone, Barbara Carlucci, Andrea Castriotti, Livio Catozzi, Giulia Chiappini, Nicola Cillara, Matteo Cinquepalmi, Enrico Coletta, Gaia Colletti, Luigi Eduardo Conte, Sophia Costacurta, Antonio Costanzo, Luciano Curella, Carmen Cutolo, Fabrizio D’Acapito, Chiara D’Alterio, Giuliano D’Onghia, Federica De Franco, Renato De Martino, Paola De Nardi, Giovanni De Nobili, Giuseppe De Ruggieri, Matteo Desio, Agnese Dezi, Giuseppe Lorenzo Di Giulio, Giulia Di Lieto, Marcello Di Martino, Giovanna Di Meo, Giulia Di Raimondo, Tommaso Dominioni, Claudia Donello, Miriam Attalla El Halabieh, Tal Deborah Engel, Lorenzo Epis, Anna Esposito, Anna Falasca, Giacinto Falco, Agostino Fernicola, Francesco Ferrara, Davide Ferrari, Lorenzo Ferri, Enrico Fischetti, Gianluca Fiumara, Laura Fortuna, Antonio Franzese, Martina Fricano, Gaetano Gallo, Alessia Galvano, Tiziana Garritano, Patrizia Alba Gentile, Giulia Germiniasi, Marco Giacometti, Mauro Giambusso, Andrea Gioffré, Gennaro Giovine, Giuseppe Giuliani, Alice Gori, Lorenzo Gozzini, Giulia Grassi, Antonella Grasso, Serena Guarriello, Maryam Hosseinpour, Ilda Hoxhaj, Alessandro Iacomino, Roberta Iadarola, Maria Iannello, Eva Iannuzzi, Luca Improta, Luca Ippolito, Roberta La Mendola, Annarita Libia, Gabriella Lionetto, Antonio Luberto, Fabrizio Luca, Claudio Luciani, Michele Manara, Serena Mantova, Chiara Marafante, Paolo Maresca, Giancarlo Maresca, Martina Marrelli, Patrizia Marsanic, Matteo Mascherini, Alberto Massocco, Manuela Mastronardi, Marco Domenico Mazza, Gennaro Mazzarella, Gennaro Melone, Paolo Enrico Meneghesso, Valentina Messina, Valentina Miacci, Flavio Milana, Margherita Minghetti, Marica Mirabella, Rosario Minà, Perla Molica, Serena Molica, Federico Morabito, Marika Morabito, Andrea Morini, Rossella Moscatiello, Edoardo Mosciatti, Marco Nicolazzi, Stefania Nigro, Cecilia Orsini, Chiara Pagnoni, Giuseppe Palomba, Elisa Paoluzzi Tomada, Vincenzo Papagni, Roberto Passa, Carola Perinotti, Bruno Perotti, Teresa Perra, Giovanni Piazza, Sara Pollesel, Gianmario Edoardo Poto, Silvia Puddu, Emanuela Querci, Valeria Quintodei, Serena Ragonici, Mario Rampa, Emanuele Rausa, Luca Resca, Valerio Rinaldi, Luca Risi, Nicola Rocco, Fausto Rosa, Leonardo Rossi, Edoardo Saladino, Giacomo Salina, Anna Sanfilippo, Pietro Santocchi, Matteo Santoliquido, Paolina Saullo, Stefania Saverino, Valentina Sbacco, Lorenzo Scardina, Andrea Scardino, Alessia Scarton, Federica Scolari, Alessandra Scotto di Uccio, Giuseppe Sena, Rosina Siciliano, Leandro Siragusa, Alessandro Soave, Marco Summa, Flavia Taglioni, Giorgio Talamo, Francesco Taliente, Marsia Tancredi, Silvia Tedesco, Ilaria Tersigni, Flavio Tirelli, Antonio Toesca, Giovanni Tomasicchio, Beatrice Torre, Irene Tucceri Cimini, Alessio Vagliasindi, Marina Valente, Mariafelicia Valeriani, Angelo Maria Velardi, Alessandro Veltri, Luca Ventrone, Tommaso Violante, Mario Gaetano Visaloco, Davide Zattoni, Iris Zoto, Arcangelo Picciariello

**Affiliations:** 1 Department of Translational and Precision Medicine, Sapienza University of Rome, Viale del Policlinico 155, 00161 Rome, Italy; 2 Surgery Unit, Department of Surgical Sciences, University of Cagliari, 09100 Cagliari, Italy; 3 UOC Chirurgia Colorettale, Fondazione Policlinico Universitario Campus Bio-Medico di Roma, Via Alvaro del Portillo, 21, 00128 Rome, Italy; 4 Unit of Surgical Oncology, Department of Medicine Surgery and Neuroscience, Azienda Universitaria Ospedaliera Senese, University of Siena, Viale Mario Bracci 16, 53100 Siena, Italy; 5 Surgical Unit of Peritoneum and Retroperitoneum Surgery, Fondazione Policlinico Universitario Agostino Gemelli IRCCS, 00168 Rome, Italy; 6 Catholic University of the Sacred Heart, Rome, Italy; 7 Centro Di Riferimento Oncologico Di Aviano (CRO), IRCCS SOC Chirurgia Oncologica del Seno, Aviano, Italy; 8 “Federico II” University Hospital, Via Pansini 5, 80131 Naples, Italy; 9 Departmant of Human Patology, Messina University Medical School Hospital, Messina, Italy; 10 Department of Medical Surgical Science and Translational Medicine, Sant’Andrea University Hospital, Sapienza University of Rome, 00185 Rome, Italy; 11 Department of Oncology, Division of Surgical Oncology and Digestive Surgery, San Luigi University Hospital, University of Turin, 10043 Turin, Italy; 12 From the Chirurgia Generale ed Epatobiliare, Azienda Ospedaliera Universitaria Integrata di Verona, Università degli Studi di Verona, Verona, Italy; 13 Peritoneal Surface Malignancies Unit, Fondazione IRCCS Istituto Nazionale dei Tumori di Milano, Milan, Italy; 14 Department of Experimental Medicine, University of Salento, Lecce, Italy

**Keywords:** Non-technical skills, Soft skills, Formal education, Surgical education, Structured curriculum, Patient care

## Abstract

Surgical training programs primarily emphasize technical skills for diagnosis and treatment. However, communication and interpersonal skills are equally essential for residents. Trainees often face sensitive situations, such as delivering bad news and discussing end-of-life decisions and lack of communications skills could result in a negative impact on patients and malpractice claims. Despite their importance, communication skills training is often underrepresented in surgical education. This study aimed to explore the extent to which such training is offered during surgical residency in Italy, and to investigate how surgical trainees and young surgeons perceive their own communication competence and confidence, particularly in emotionally demanding scenarios. The “COSTRUIRE” (COmmunication Skills TRaining in sUrgIcal REsidency) survey, conducted from July to September 2024 by the Young Group of the Italian Society of Surgical Oncology, utilized a 30-item online questionnaire to gather data on participants’ communication experiences, emotional experiences, burnout risk, and training preferences. The responses were analyzed in accordance with the CHERRIES guidelines. A total of 189 participants met the inclusion criteria, including general surgery residents (61.4%) and early-career surgeons. Most respondents (85.2%) reported having communicated a difficult diagnosis without supervision during training. While participants rated their communication skills positively, over 70% expressed a desire to have handled these conversations differently, often citing the need for better tools or support. Notably, 73.9% reported learning communication informally by observing mentors. Only 7.9% received formal training during residency, despite 91% recognizing its importance. Emotional impact was significant, with high levels of emotional involvement but also early signs of burnout. The COSTRUIRE survey identified the importance of structured communication skills training in Italian surgical residency. Addressing this gap may contribute to improving self-perceived competence and confidence in managing emotionally challenging clinical interactions.

**Supplementary Information:**

The online version contains supplementary material available at 10.1007/s13304-026-02557-2.

## Introduction

Practicing surgical skills is one of the most essential tasks for trainees enrolled in the national surgical training program. Nonetheless, several studies showed that developing communication and interpersonal skills is also a valuable component of surgical training [1–6]. Non-technical skills include communication, teamwork, situational awareness, leadership, and decision-making—all significant for patient safety and effective functioning in high-stakes clinical environments [2, 3]. These skills allow surgeons to coordinate complex multidisciplinary care, manage critical situations under pressure, and build trusting relationships with patients and their families. This need was particularly evident during the COVID-19 pandemic, which exponentially underscored the importance of developing “non-technical skills” [7, 8]. In fact, surgical trainees can be confronted with challenging situations, such as delivering bad news or discussing end-of-life issues [9]. Furthermore, they are required to perform such sensitive tasks in various environments, from outpatient clinics to intensive care units [1]. A lack of adequate communication and “non-technical skills” may lead to malpractice claims [6] and surgical adverse events [9] that could have been prevented with appropriate training.

Formal training in communication during residency is more official than practical, and trainees frequently feel unprepared to hold challenging conversations [2]. A prospective study involving 44 University of Connecticut general surgery residents reported that without communication skills, even the best surgical training would be rendered ineffective [5].

The importance of communication skills training has been well established in other specialties, such as oncology and critical care. However, these competencies are not yet systematically addressed in surgical education [6]. As of today, in Italy there are no national guidelines or standardized curricula and include them. Exposure to those themes is variable and often relies on the hospital’s initiative. The objective of this survey is to examine the extent of communication training available and to understand how residents and young surgeons view their readiness to navigate complex interpersonal dynamics in surgical settings.

## Material and methods

A 30-item questionnaire was distributed online between July to September 2024 via the official channel of the Italian Society of Surgical Oncology (SICO), including the society’s newsletter and mailing list.

SICO is a national scientific society that includes general surgeons, surgical oncologists, and trainees with an interest in oncologic surgery, many of whom are affiliated with academic hospitals and university departments.

A brief introduction outlined the purpose of the survey to all participants, and they were invited to voluntarily consent to a privacy policy. The survey targeted residents and early-career specialists in general surgery. Inclusion criteria were surgical residents enrolled in accredited Italian training programs and early-career surgeons under the age of 40 who were members or affiliates of the SICO.

After a comprehensive review of the literature using keywords such as “communication, skills, surgical training, non-technical skills, soft skills, medical education, doctor and patient relations,” a questionnaire was developed and administered in Italian. The survey focused on patient-centered communication, specifically the ability of trainees to handle emotionally complex interactions.

**Fig. 1:**
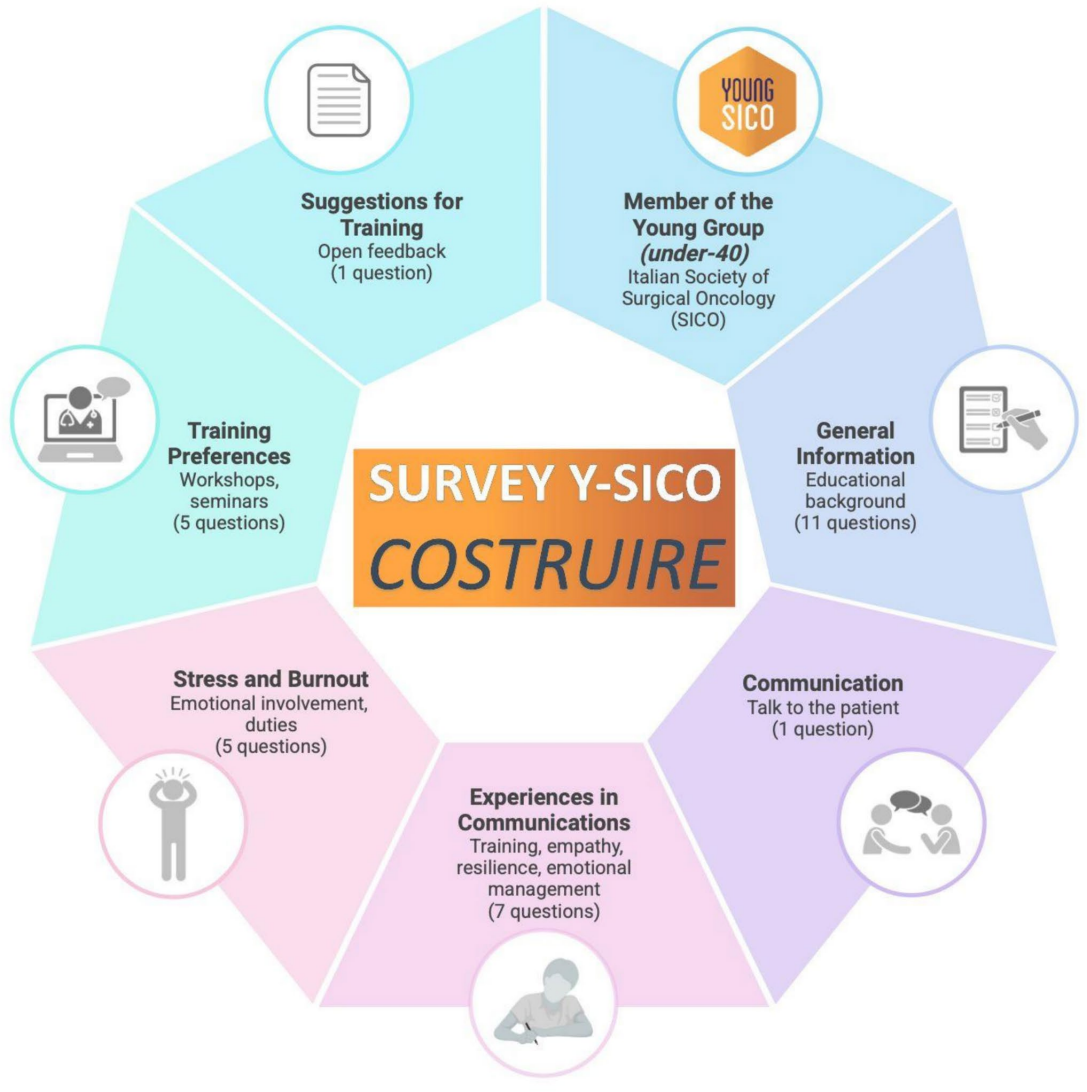
Survey Design: An Overview of Origin and Sections. This figure illustrates the group that inspired the survey’s design and outlines its key components. “Y-SICO”: Young-Italian Society of Surgical Oncology; “COSTRUIRE”: COmmunication Skills TRaining in sUrgIcal REsidency

The survey acronym “COSTRUIRE” (COmmunication Skills TRaining in sUrgIcal REsidency) reflects the constructive purpose to improve communication training for surgical trainees in Italy, rather than criticizing existing practices (Fig. 1).

The questionnaire was developed by R.M. and A.P., in collaboration with the Young SICO Board, and reviewed by a recognized expert in medical pedagogy and innovative teaching methods in Italy, with specific expertise in surgical education. The survey was reviewed and approved by the Presidents of the Italian Society of Surgical Oncology (SICO). To assess clarity and content relevance, the questionnaire was pilot-tested with a small group of five surgical residents. Their feedback led to minor adjustments before final distribution.

The questionnaire was specifically developed for the purpose of this study and was not formally validated. Therefore, the results reflect self-perceived communication skills and emotional experiences rather than objectively measured competencies.

Data was collected through a 30-item online questionnaire (Appendices A and B) created using Google Forms (Google LLC, Mountain View, California, US), and divided into six sections.

1. *General Information (11 questions)*: This section collected demographic and training background data. It included 3 initial questions about age and professional status, with the final question asking whether the respondent was a resident or a practicing surgeon. Based on this response, participants were directed to an additional set of 4 questions exploring their educational background, such as the university attended for their medical degree and specialty.
2. *Communication (1 question)*: This question assessed whether participants had ever communicated a difficult diagnosis (e.g., cancer, end-of-life discussion, medical/surgical error) by themselves, without support from a tutor or senior professional. Those who answered affirmatively were directed to subsequent questions examining their experiences in greater detail (in  "Results" section– Experiences in Communication – and  "Discussion" section– Stress and Burnout). Participants who responded negatively were instead directed to  "Conclusion" section (Training Preferences) and Section 6 (Suggestions for Training), which explored their expectations, and proposals regarding communication education.
3. *Experiences in Communication (7 questions)*: This section inquired about personal experiences in delivering bad news and the emotional impact of these interactions. Several questions were designed as multiple-choice to gain a wider range of participants’ perspectives. Communication skills were assessed through two distinct self-evaluation items. One asked participants to rate, on a 5-point Likert scale (1 = very poor; 5 = excellent), their communication abilities in emotionally complex situations. It also asked them to assess how clear and empathic they believed they were, and the extent to which they felt the patient had understood them.

A second item asked respondents to provide an overall self-assessment of their communication competence using a 10-point numeric scale (1 = not competent at all; 10 = fully competent).

4. *Stress and Burnout (5 questions)*: Assessed emotional involvement, exhaustion, and perceived burnout related to communication tasks. “Emotional involvement” referred to the degree of personal emotional engagement experienced during these interactions.
5. *Training Preferences (5 questions)*: Explored interest in additional communication training and format preferences, such as workshops, role-playing exercises, or online seminars.
6. *Suggestions for Training (1 question)*: Participants could provide open-ended feedback and recommendations for improving communication training within surgical programs.

The survey results were presented in accordance with the Checklist for Reporting Results of Internet ESurveys (CHERRIES) guidelines [10].

### Participant recruitment and response rate

At the time of survey dissemination (September 2024), the Young SICO group comprised 222 officially registered members under the age of 40. The survey was distributed via the national newsletter of the Italian Society of Surgical Oncology (SICO), allowing it to reach a broader audience beyond Young SICO members. A total of 212 responses were collected. Although the survey was specifically designed for surgical trainees and early-career surgeons under 40, its open distribution resulted in a small number of responses from older participants. In line with the predefined inclusion criteria, 23 responses from individuals over 40 were excluded. The final analytic sample included 189 respondents, representing 85.1% of all officially registered Young SICO members.

### Data analysis

Survey responses were analyzed using Google Sheets (Google LLC, Mountain View, California, US). Descriptive statistics, including frequencies and percentages, were used; no inferential analyses were performed. The survey incorporated branching logic to collect targeted responses based on participants’ roles and experiences, allowing for a more tailored and context-specific analysis. The questionnaire did not include open-ended questions designed for qualitative analysis; however, participants had the option to leave free text comments at the end. These comments were reviewed to capture general impressions but were not formally coded or analyzed.

### Ethical considerations

According to national regulations, formal approval from an ethics committee was not required for this study, as it was based on a voluntary survey and did not involve the collection of sensitive personal data. While the survey requested participants’ names solely for the purpose of recognizing contributors in the “collaborative group,” confidentiality was strictly maintained. The initial description of the survey clearly informed participants about the voluntary nature of the study and included a specific question asking for their consent to participate. Personal data was handled in accordance with data protection regulations and used exclusively for attribution. All responses were processed and stored securely, and identifying information was not linked to the survey data during analysis.

## Results

### Demographic characteristics

A total of 189 participants met the inclusion criteria and were included in the analysis. Among them, 116 (61.4%) were current General Surgery residents, while 73 (38.6%) were early-career surgeons with a mean of 2.81 years and a median of 2.0 years since completing residency, as shown in Table 1. The sample comprised 95 males and 94 females from surgical centers across Italy. The mean age of participants was 31.88 years (range: 25–40). Based on participants’ initial response regarding their status, the survey used branching logic to design subsequent questions accordingly.

**Table 1:** Demographic characteristics of participants

Characteristics of participants		
	n	%
Total n. of participants	189	100.0
Gender		
Males	95	50.3
Females	94	49.7
Residency		
Current residents	116	61.4
1st year	7	6.0
2nd year	23	19.8
3rd year	33	28.4
4rd year	34	29.3
5th year	19	16.4
General surgeons (age ≤ 40y)		
Early-career surgeons (within 3-y)	50	26.5
Surgeons (for more than 3-y)	23	12.2

This table summarizes the key demographic characteristics of the survey participants, including age, gender, and surgical experience. N.: number; y: years

Among the 73 respondents who had completed their residency, 40 (54.8%; 26.5% of the total sample) completed both their medical degree and residency at the same university, while 33 (45.2%; 17.5% of the total sample) pursued their residency at a different institution (full distribution available in Supplementary Table S1).

Regarding the 116 current residents, the distribution by year of training was: 7 (6.0%) in their first year, 23 (19.8%) in the second, 33 (28.4%) in the third, 34 (29.3%) in the fourth, and 19 (16.4%) in the fifth year. Half of these residents (58, 50%) remained at the same university where they earned their medical degree, while the other 58 (50%) were training at a different institution (see Supplementary Table S2 for the full list).

### Communication experiences

**Fig. 2:**
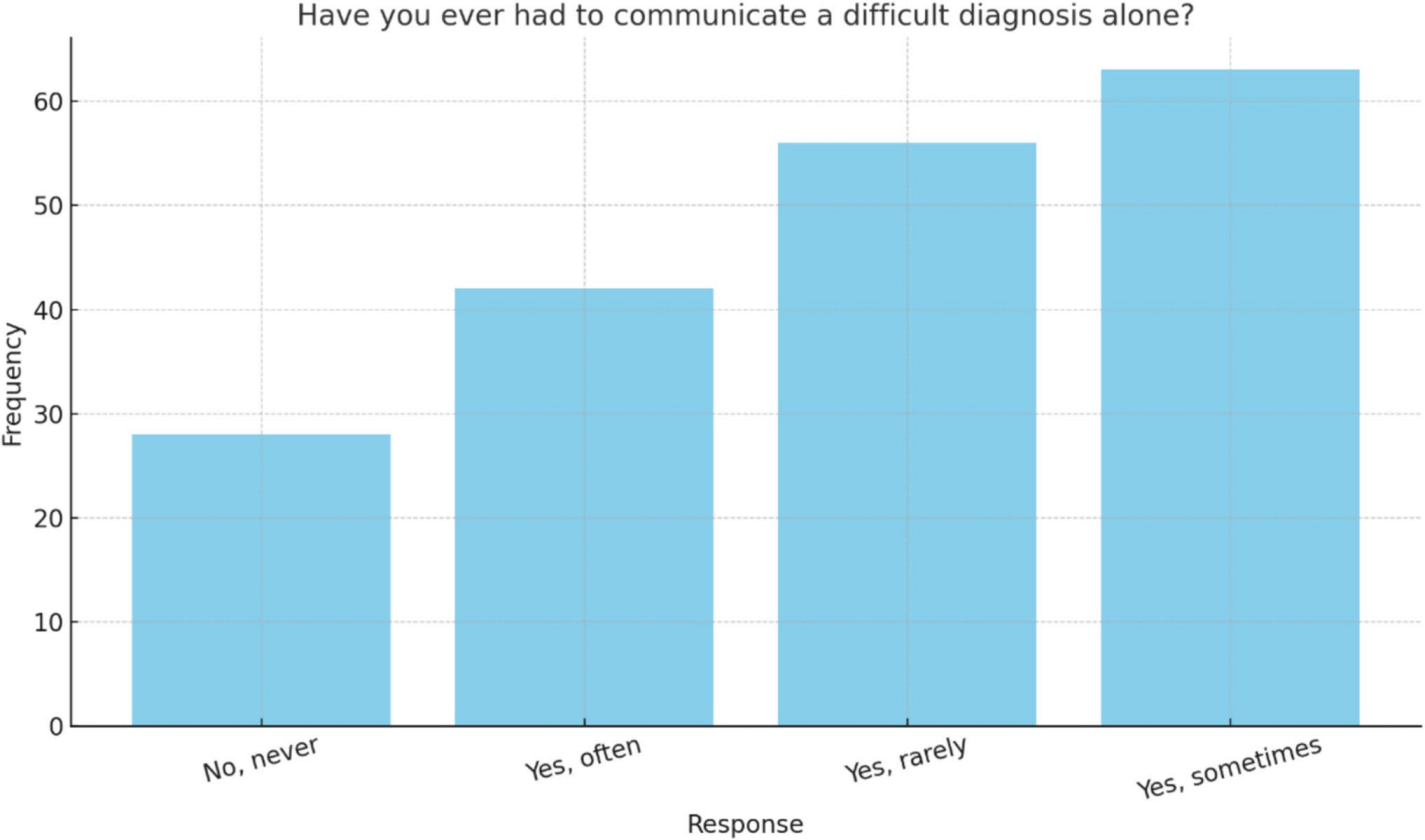
Frequency of communication of a difficult diagnosis without supervision or senior support among surgical trainees. Distribution of responses to Question 13: “During your training in General Surgery, have you ever had to communicate a difficult diagnosis (e.g., cancer, end-of-life, medical/surgical error) alone, without support from a tutor or more experienced professional?”. Most participants reported having encountered such situations at least occasionally, with 85.2% responding “Yes” (either often, sometimes, or rarely)

Overall, 161 participants (85.2%) reported having independently communicated difficult diagnoses, such as cancer or end-of-life discussions, at least once during their training (Question 13). Among them, 42 (22.2%) did so frequently, 63 (33.3%) occasionally, 56 (29.6%) rarely, while 28 (14.8%) had never faced this situation (see Fig. 2, Table 2).

**Table 2:** Past communication experiences

Past communication experience		
	n	%
Q. n. 13—Independent difficult diagnoses communication (n = 189)		
Frequently	42	22.2
Occasionally	63	33.3
Rarely	56	29.6
Never	28	14.8
Q. n. 14 – Emotional state while communicating a difficult diagnosis* (n = 161)		
Feeling calm	52	32.3
Feeling confident and in control	88	54.7
Frustrated from being alone	30	18.6
Scared	11	6.8
Insecure (I didn’t know how to express myself)	18	11.2
Embarrassed (I didn’t want to be in that situation “without tools”)	34	21.1
Ashamed in front of the patient and family	3	1.9

The table provides the results of the Survey about the Experiences of Participants in Communicating Difficult Diagnoses Without a Mentor. Legend: N.: number. * Note: Multiple responses per participant were allowed

**Fig. 3:**
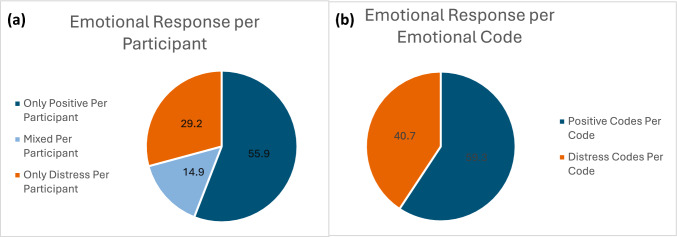
Emotional Reactions to Unsupervised Communication of Difficult Diagnoses. The left pie chart (**a**) illustrates the distribution of emotional responses among participants (Question 14), categorized as only positive (e.g., feeling confident and in control, or calm), mixed (both positive and distress-related), or only distress-related (e.g., embarrassment, insecurity, or scare). The right pie chart (**b**) presents the distribution of the total number of emotional codes selected, reflecting the overall prevalence of positive versus distress-related emotions, regardless of whether multiple codes were selected by the same participant

When reflecting on their emotional state during unsupervised communication of difficult diagnoses (Question 14), 55.9% of participants reported exclusively constructive emotions (e.g., calmness, confidence), 14.9% reported mixed emotions, and 29.2% reported solely discomforting emotions (e.g., embarrassment, insecurity) (Fig. 3a).

Across all selected emotional responses (multiple allowed), 59.3% were constructive, and 40.7% indicated distress or discomfort (Fig. 3b).

Among the 161 participants who had previously communicated a difficult diagnosis alone, the most frequently reported emotional reactions were:

- Feeling confident and in control (88 participants, 54.7%),
- Feeling calm (52 participants, 32.3%),
- Embarrassed due to lack of tools (34 participants, 21.1%),
- Frustrated from being alone (30 participants, 18.6%) (Table 2).

These findings show that a notable portion of respondents, despite managing difficult communications with apparent calm, still experienced adverse emotional responses, highlighting the need for adequate emotional preparation and support during training. To investigate whether emotional reactions differ by frequency of independently delivering difficult diagnoses, we compared emotional state distributions across three subgroups: frequent, occasional, and rare communicators (Questions 14 and 13).

Participants with frequent exposure (“Yes, often”) predominantly reported constructive emotions, with 73.8% feeling calm and confident and 31.0% feeling tranquil. Conversely, those with rare exposure exhibited higher rates of distress, including insecurity (21.4%), frustration (21.4%), embarrassment (19.6%), and fear (10.7%). The “Yes, sometimes” group demonstrated a wider emotional spectrum, with 23.8% reporting embarrassment and a mix of positive and negative feelings (Table 3).

**Table 3:** Distribution of emotional reaction according to exposure frequency to unsupervised difficult conversations

Emotional Reaction (Q. n. 14)	Frequency of unsupervised difficult communication (Q. n. 13)		
	Yes, often n (%)	Yes, rarely n (%)	Yes, sometimes n (%)
Feeling confident and in control	31 (73.8)	27 (48.2)	30 (47.6)
Embarrassed (I didn’t want to be in that situation “without tools”)	8 (19)	11 (19.6)	15 (23.8)
Insecure (I didn’t know how to express myself)	2 (4.8)	12 (21.4)	4 (6.3)
Frustrated from being alone	10 (23.8)	12 (21.4)	8 (12.7)
Feeling scared	1 (2.4)	6 (10.7)	4 (6.3)
Feeling calm	13 (31)	15 (26.8)	24 (38.1)
Ashamed in front of the patient and family	0	2 (3.6)	1 (1.6)

This table shows the percentage (%) and absolute number (n) of participants who reported each emotional reaction (collected in Question 14), grouped by the frequency with which they had to communicate a difficult diagnosis without supervision (Question 13). Legend: % = Percentage of participants within each frequency group who selected that emotional reaction; n = Absolute number of participants within each group selecting the reaction

These results suggest that increased exposure may foster a sense of confidence, while limited or sporadic exposure may be associated with higher emotional vulnerability (Tables 4, 5).

**Table 4:** Preferred approach to communicating a difficult diagnosis

Response option	Frequency	Percentage
Alone but with more confidence/tools	47	29.2
I communicated the way I wished to	45	28
Knowing how to manage the interlocutor’s emotions	33	20.5
With support of the tutor	25	15.5
Knowing how to structure the communication	11	6.8

Distribution of responses to Question 19, which asked participants how they would have preferred to handle the communication of a difficult diagnosis

**Table 5:** Previous exposure to communication training

Response option	Frequency	Percentage
Observed tutors/colleagues to form my own idea	119	73.9
No specific tools provided	21	13
Self-initiated study (literature, web, courses)	6	3.7
Received formal training during medical school	15	9.3

Distribution of responses to Question 20, which investigated participants’ prior exposure to communication training

Approximately three-quarters of respondents (73.9%) reported that informal observation of tutors or colleagues represented their only source of training

### Perceived preparedness and training

A total of 161 respondents with prior experience in difficult conversations completed items on communication skills and emotional impact (In  "Results" and "Discussion" sections). Those reporting no such experience were redirected to  "Conclusion" and Section 6, which focused on training preferences (see Methods).

Participants rated their communicative effectiveness following difficult diagnoses across three dimensions: clarity, patient understanding, and empathy (Questions 15–17), using a 5-point Likert scale (1 = low, 5 = high).

**Fig. 4:**
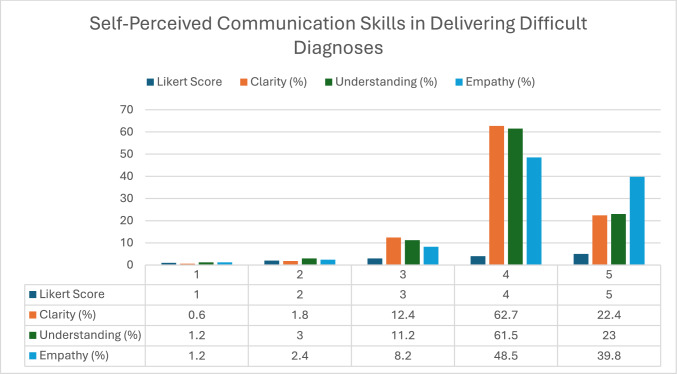
Self-Perceived Communication Skills in Delivering Difficult Diagnoses. Distribution of responses to Questions 15–17, assessing participants’ self-perceived communication skills when delivering difficult diagnoses without supervision. Responses were given on a 5-point Likert scale (1 = very low, 5 = very high)

Results indicated generally high self-assessments: clarity was rated 4 or 5 by 85.1% of respondents (62.7% scored 4; 22.4% scored 5), patient understanding by 84.5% (61.5% scored 4; 23.0% scored 5), and empathy by 88.3% (48.5% scored 4; 39.8% scored 5) (Fig. 4). Low ratings (1 or 2) were rare, especially for clarity (0.6%) and empathy (1.2%).

Participants rated their overall satisfaction with their communication during difficult encounters on a 10-point Likert scale (1 = very low, 10 = very high; Question 18). The 10-point scale was chosen to provide greater nuance compared to the 5-point scales used for clarity, empathy, and patient understanding.

**Fig. 5:**
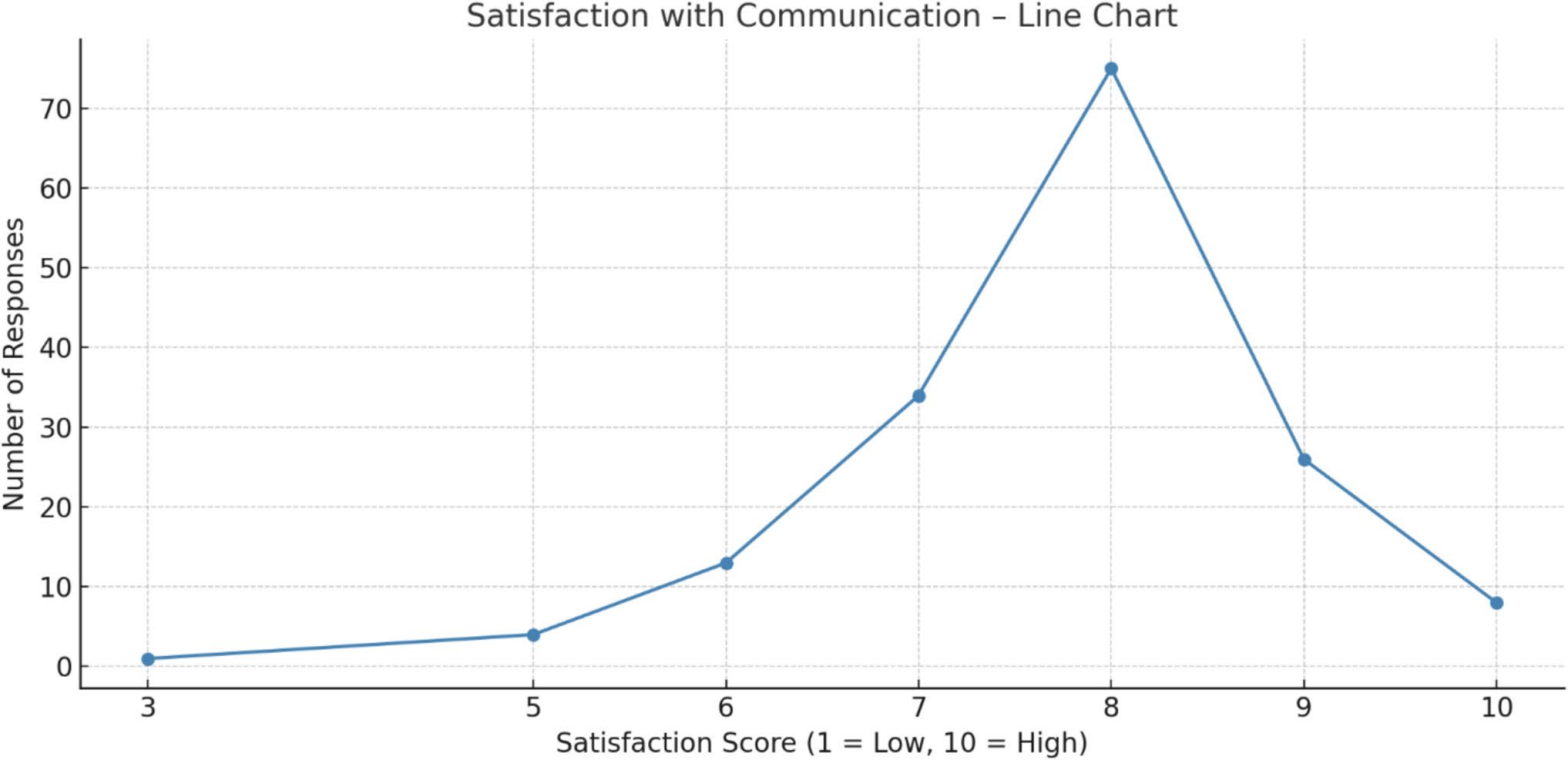
Self-Reported Satisfaction with Communication During a Difficult Encounter. Distribution of responses to Question 18, in which participants rated their overall satisfaction with how they communicated during a difficult clinical conversation, using a 10-point Likert scale (1 = very low, 10 = very high). The distribution shows a distinct peak at score 8 (46.6%), with additional concentrations at scores 7 (21.1%) and 9 (16.2%). Only 5.0% of respondents rated their satisfaction at the highest score (10), suggesting that although participants generally felt confident, many still recognized areas for improvement in their communication skills

As shown in Fig. 5, responses peaked at score 8 (46.6%), followed by 7 (21.1%) and 9 (16.2%). Ratings below 6 were rare, and only 5.0% reported full satisfaction (score 10). These findings indicate generally high satisfaction, while suggesting most participants recognized room for improvement. The modest selection of the highest score may reflect awareness of the emotional and technical challenges involved in delivering difficult news.

Regarding communication challenges (Question 19), only 28.0% of participants felt they managed the situation as they wished. The majority expressed a need for greater support or preparation: 29.2% desired more confidence and communication tools to handle the situation independently, 20.5% sought improved skills for managing the emotional aspects, 15.5% wanted tutor support, and 6.8% wished for guidance on structuring the communication (Table 4).

These findings correspond with the self-assessed satisfaction ratings (Question 18), where most participants scored their performance between 7 and 9 out of 10, indicating moderate satisfaction but also room for improvement. The fact that over 70% preferred alternative approaches underscores the need for formal training addressing both message delivery and emotional competence to foster autonomy.

Regarding training (Question n. 20), approximately three-quarters of participants (73.9%) reported that informal observation of tutors or other professionals was their sole exposure to communication training. Only 9.3% had received formal instruction during medical school, while 13% indicated they had not received any specific training (Table 5).

**Fig. 6:**
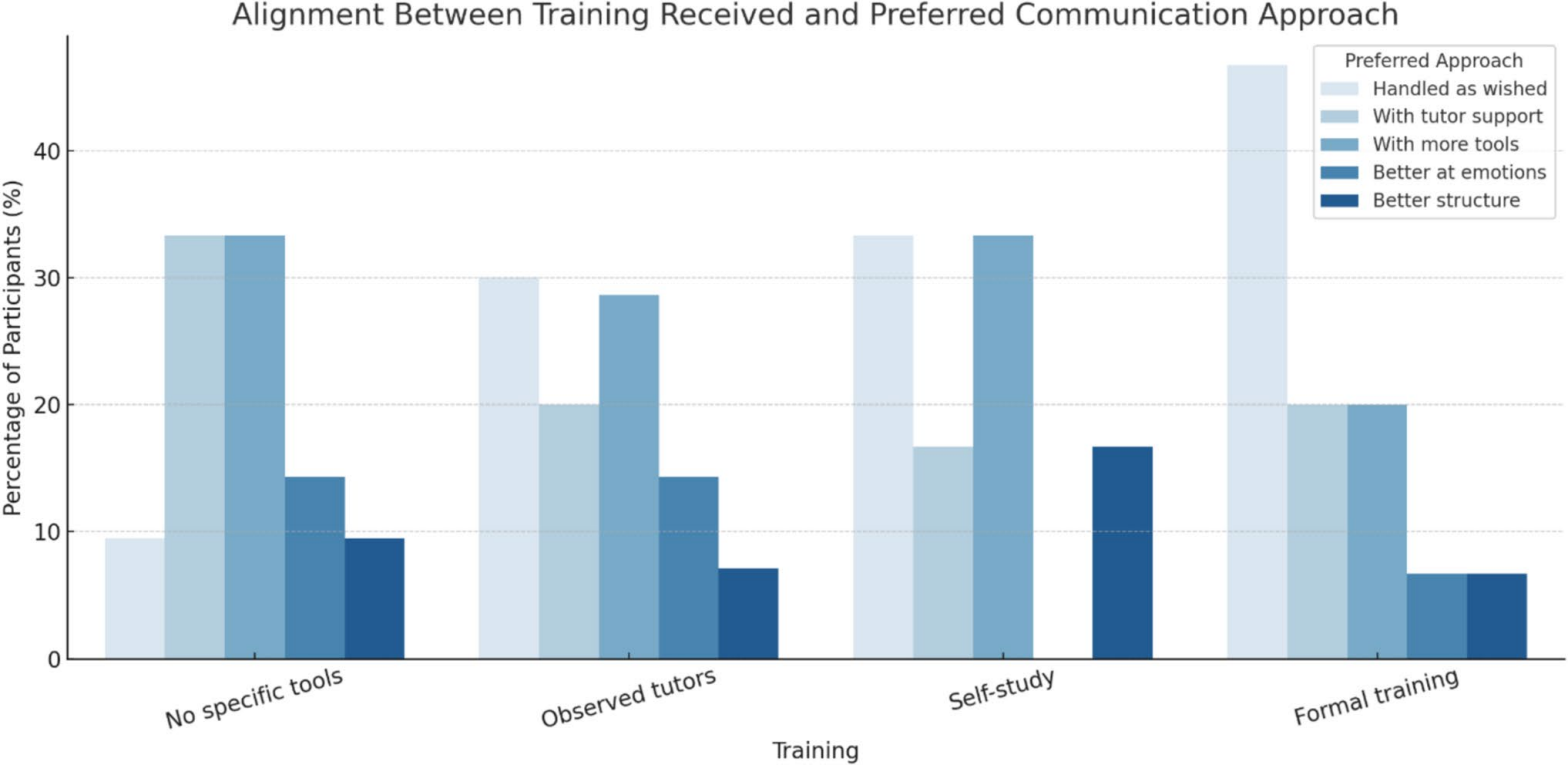
Alignment Between Received Training and Preferred Approach to Difficult Communication. Comparison between the type of training received (Question 20) and participants’ preferred way of handling the communication (Question 19). The highest proportion of participants who felt they had managed the conversation as they wished was observed among those who had received formal training during medical school (46.7%). Conversely, among those who had no specific tools, only 9.5% expressed satisfaction, and most wished for either more autonomy or supervisory support

When comparing the communication tools participants reported receiving with their preferred approach to managing conversations, several patterns emerged. Among those without specific tools (13.0%), only 9.5% felt they handled the conversation as they wished, while 66.6% expressed a need for either tutor support or greater autonomy with improved tools (Fig. 6). Participants who had only observed tutors or colleagues (73.9%) were more divided: nearly 30% felt they managed the conversation satisfactorily, but 28.6% wished they had better resources to handle it independently. Notably, the highest proportion of satisfaction (46.7%) was reported by those who had received formal communication training during medical school, suggesting that such training may be crucial for elevating residents’ communicative confidence and autonomy.

### Training

While 91.0% of participants rated communication training as a crucial educational expectation for General Surgery residents (scores 4 or 5 on a 5-point Likert scale; Question 22), only 7.9% reported its actual availability within their residency programs (Question 21). Additionally, 66.1% indicated that no formal communication training was provided, and 25.9% were unaware of its presence (Table 6). This perception was further corroborated by responses to Question 23, where 77.3% of participants assigned a rating between 8 and 10 on a 10-point scale for the importance of communication skills in the overall training of a General Surgery Specialist, with 28.6% selecting the maximum score of 10 and only 8.5% rating it 6 or below.

**Table 6:** Communication training

Communication training		
	n	%
Residency training (n = 189)		
No formal training	125	66.1
Specific training	15	7.9
Unknown	49	25.9
Value given to communication skills in surgical training (n = 189)		
Essential (5)	118	62.4
High-level (4)	54	28.6
Moderate (3)	16	8.5
Low-grade (2)	1	0.5
Not important (1)	0	0

Participants’ Responses to Training Received on Patient Communication and the Value They Place on Acquiring This Skill (Question n. 21 e 22). N.: number

When asked about including a module on high-quality communication in surgical oncology residency programs (Question 24), there was strong support for formal training: 45% of respondents assigned the highest rating [5], with an additional 37% selecting 4. Only 3.2% expressed limited interest (score 2), and none chose 1.

In open-ended responses regarding preferred training methods (Question 25), participants could select multiple options. A majority (73%, n = 138) advocated for involvement of professionals specifically trained in communication to facilitate skill development. Other suggestions included role-playing exercises with real-world scenarios (34.9%, n = 66), webinars (24.9%, n = 47), and methodical lessons on communication (19.6%, n = 37).

### Emotional impact and burnout

**Fig. 7:**
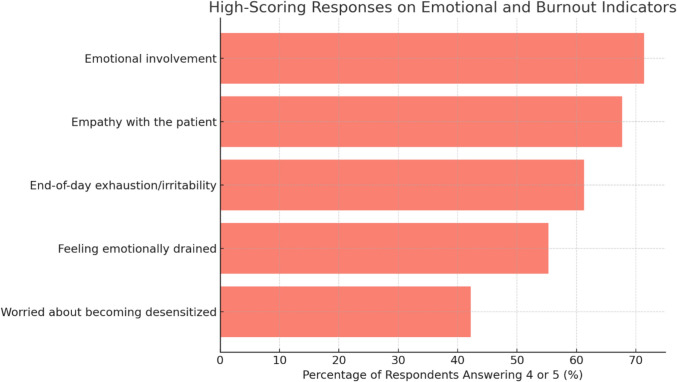
Emotional impact and burnout. Questions 28–30—Participants’ Emotional Experiences in Patient Communication and Their Perceptions of Potential Burnout

To assess residents’ emotional experiences and potential burnout indicators, participants rated five statements on emotional involvement, empathy, and stress using a 5-point Likert scale (1 = not at all, 5 = very much). Figure 7 presents the proportion endorsing high scores (4 or 5) for each item. Results revealed a dual emotional pattern: high emotional engagement, 71.4% reported feeling involved in their work and 67.7% found empathizing with patients easy, alongside notable signs of emotional strain, with 61.2% experiencing end-of-day exhaustion and irritability, 55.3% feeling emotionally consumed, and 42.2% concerned about potential desensitization over time.

## Discussion

This study aimed to comprehensively describe the current state of communication skills training for surgical residents and early-career surgeons within the Italian Society of Surgical Oncology (SICO).

Our findings revealed a substantial gap in formal instruction, underscoring the importance of embedding communication training into the core curriculum of surgical education [11, 12]. Notably, 85.2% of participants said they had to communicate a difficult diagnosis on their own during training, while only 14.8% had never encountered this situation during their residency.

Although participants generally reported high self-perceived communication effectiveness, most respondents described situations in which they felt emotionally exposed, underprepared, or unsupported, particularly when having to manage these conversations by themselves.

While repeated exposure appeared to be associated with greater emotional confidence, less experienced residents reported higher levels of discomfort, including embarrassment, insecurity, and nervousness.

Communication in healthcare, particularly oncology, involves a complex interplay of emotions, expectations, and motivations as well as requiring healthcare providers to act responsibly given the asymmetry in roles [13, 14]. Greenberg et al. [15] highlighted the benefits of improved communication protocols and greater engagement with attending surgeons to prevent errors and improve patient outcomes. In fact, effective communication is a core competency in surgical training, essential for patient empowerment and shared decision-making (SDM) [16–18]. SDM is increasingly acknowledged as a pivotal element of patient-centered care, with positive effects on both surgeon and patient satisfaction, reduced conflict and anxiety, as well as better understanding of surgical procedures, and a lower risk of malpractice [19, 20]. Despite these benefits, SDM in surgery is under-researched, with few published studies, focusing on plastic surgery and elective operations [16, 18].

SDM is considered especially important in surgical settings due to the often irreversible nature of procedures and their potential to significantly alter patients’ physical functioning, directly affecting outcomes and satisfaction [16, 17, 20].

In the last decades patient advocacy groups have worked with healthcare professionals to drive advancements in research and treatment [21–23]. Moreover, patient-centered care emphasizes understanding individual patient needs and preferences, which leads to better health outcomes [20, 24].

Data from the COSTRUIRE survey made evident that systematic communication training is largely absent from Italian surgical residency programs.

Though most participants rated themselves as capable of being clear, empathic, and understanding, more than 70% indicated they would have preferred to handle the conversation differently. Many wished for better tools, more organized training, or the support of a tutor, suggesting a clear gap between perceived adequacy and actual preparation. Furthermore, 73.9% of participants reported learning to communicate difficult diagnoses by observing their mentors or colleagues.

Participants who had received formal communication training during medical school reported the highest levels of self-competence, highlighting the positive impact of early, methodical education.

The integration of formal communication training into surgical education as a foundational requirement ensures consistency and depth in teaching communicative competence across clinical contexts.

Our survey also revealed a mismatch between expectations and training opportunities: while 91% of participants considered communication training essential, only 7.9% reported having access to formal teaching during residency. This training deficit reflects a broader, systemic underestimation of communication as a fundamental surgical skill and calls for a more assimilated approach in teaching.

The emotional impact of these experiences should not be overlooked. A significant portion of participants reported signs of emotional strain, including exhaustion, irritability, and concern about becoming desensitized. One-third of the survey respondents found the process stressful [25]. These findings echo existing literature on the emotional burden of clinical training and reinforce the need for a support system aimed at promoting emotional resilience and well-being in surgical education.

Feelings of fear, blame, negative emotional reactions of patients and their relatives, are common [26, 27]. These findings align with existing literature showing that insufficient development of non-technical skills contributes to higher rates of burnout among physicians and trainees [28, 29]. Conversely, communication training has been associated with improved emotional resilience, reduced anxiety, and stronger clinician-patient relationships [30–32].

Effective methods to improve communication skills in surgical training include a combination of lectures, role-playing, simulations, peer feedback, and briefing and debriefing tailored to surgical context. Feedback from instructors and simulated patients and scenarios were beneficial in establishing a safe setting. An example is the OncoTalk model, developed in 2002 for medical oncology fellows, aimed to address communication challenges between physicians and cancer patients [33]. In 2017, Coleman et al. adapted this model for surgical trainees (5). The SurgicalTalk curriculum involved two-hour sessions per year for surgical residents, integrated into the academic schedules, with 4–6 residents attending each session. Between July 2017 and June 2021, they held 6–7 workshops annually, with mandatory participation for all surgery residents. Participants expressed satisfaction with the curriculum, indicating its effectiveness in improving their communication skills.

As suggested by 75% of our respondents, consulting psychologists and psychotherapists during the training process could help trainees develop emotional intelligence and resilience.

In other countries, like the United Kingdom, the Royal College of Surgeons includes modules on breaking bad news and SDM within its core surgical curriculum. In the United States, communication training is a core competency in the ACGME (Accreditation Council for Graduate Medical Education) program, and simulation-based curricula have been widely applied in surgical residencies. [34, 35].

Such programs are not yet consistently implemented in Italy, and our findings suggest that surgical trainees and early-career surgeons would benefit from similar standardized approaches. Tailoring these strategies to Italian surgery schools would require institutional support, national curriculum integration, and faculty training in communication pedagogy.

Observing a mentor delivers bad news does not necessarily prepare a young resident to manage such situations independently. Just as modern surgical training has moved beyond the “see one, do one, teach one” model in the operating room [36–38], we should not expect soft skills to be acquired passively.

Several limitations of this survey should be acknowledged. First, the use of a self-reported, non-validated questionnaire may have introduced response bias, as participants’ assessments reflect perceived rather than objectively measured communication competencies. Additionally, the cross-sectional design does not allow for causal inferences about the relationship between communication training and outcomes such as burnout or patient satisfaction. The online distribution of the survey through SICO newsletter limits the generalizability of the findings, as responses were collected exclusively from members of the SICO, and may not reflect the views or experiences of surgical trainees and early-career surgeons outside this professional network.

## Conclusion

While mastery of technical skills remains crucial, it should be augmented by interpersonal abilities to navigate the complex relational aspects of surgical care. Establishing structured communication training programs could address this shortfall, cultivating surgeons who are more confident, empathetic, and resilient—ultimately shaping the next generation of surgical leaders.

## Supplementary Information

Below is the link to the electronic supplementary material.

Supplementary file1 (PDF 296 KB)

Supplementary file2 (PDF 212 KB)

Supplementary file3 (DOCX 18 KB)

Supplementary file4 (DOCX 17 KB)

Supplementary file5 (DOCX 17 KB)

## Data Availability

All data are included in the article or its supplementary materials.

## Declarations

### Prior presentation

This paper has not been presented at any scientific meeting.
