# Supplementary material for: Communication skill training in surgical residency: insights from Y-SICO (Young-Italian Society of Surgical Oncology)
Source: Updates Surg. 2026 Apr 10;78(4):1811–24. doi: 10.1007/s13304-026-02557-2 (PMC13421255; doi:10.1007/s13304-026-02557-2)
Supplement: Supplementary file 1 — Supplementary file1 (PDF 296 KB) [file 13304_2026_2557_MOESM1_ESM.pdf]

Survey Young-SICO "*COSTRUIRE*": **CO**mmunication **S**kills **TR**aining in s**U**rg**I**cal **RE**sidency

Come comunichiamo la diagnosi di cancro?

Cara/o Collega,
Cogliamo l'occasione per ringraziarTi della Tua cortese disponibilità a collaborare a questa ricerca che, ricordiamo, è promossa da Young SICO (Società Italiana di Chirurgia Oncologica).
Il questionario che Ti inviamo è di breve compilazione (ca. 5 min.) ed ha lo scopo di *fotografare* e *comprendere* quale sia il percorso formativo offerto oggi agli specializzandi e giovani neo-specialisti, con max 3 anni di esperienza lavorativa, in Chirurgia generale riguardo il tema della "*comunicazione medica*", in particolare *oncologica*.
Non esistono dunque risposte "giuste" o "sbagliate" alle domande di seguito proposte, quindi sentiTi libero di scegliere la risposta in cui Ti riconosci di più.
Ti ringraziamo anticipatamente per la collaborazione e il tempo che ci dedicherai, sottolineando come partecipando a questa survey, verrai incluso nell'*authorship* del "*Collaborative Group*" di questa ricerca.
Per questo motivo, all'inizio del questionario verranno chiesti nome e cognome, ma in seguito il sistema Ti assegnerà un numero identificativo nel rispetto della Tua privacy.
Le Tue informazioni verranno tenute strettamente riservate.
A presto!
Board Y-SICO

* Indica una domanda obbligatoria

1. Consenso informato *

Contrassegna solo un ovale.

- ( ) Acconsento
- ( ) Non acconsento

Informazioni generali

2. Nome e Cognome *

____

3. Quanti anni hai?

4. Sei attualmente un medico in formazione in Chirurgia Generale o ti sei da pochi anni specializzata/o nella stessa disciplina? *

Contrassegna solo un ovale.

- Sono un medico specializzando in Chirurgia Generale *Passa alla domanda 9.*
- Ho conseguito la specializzazione in Chirurgia Generale da meno di 3 anni *Passa alla domanda 5.*
- Ho conseguito la specializzazione in Chirurgia Generale da più di 3 anni *Passa alla domanda 5.*

Medico neo-specialista in Chirurgia Generale

5. Quale Scuola di Specializzazione in Chirurgia Generale hai frequentato?

6. Da quanti anni hai conseguito la specializzazione?

7. Hai conseguito la Laurea in Medicina e Chirurgia nella stessa sede universitaria nella quale hai conseguito la specializzazione?

Contrassegna solo un ovale.

- Sì
- No

8. In quale Università italiana hai conseguito la Laurea in Medicina e Chirurgia?

____

Passa alla domanda 13.

Medico specializzando in Chirurgia Generale

9. A quale Scuola di Specializzazione in Chirurgia Generale risulti attualmente iscritto? *

____
____
____
____
____

10. A quale anno di Scuola di Specializzazione risulti attualmente iscritto? *

Contrassegna solo un ovale.

- Primo
- Secondo
- Terzo
- Quarto
- Quinto

11. Hai conseguito la Laurea in Medicina e Chirurgia nella stessa sede universitaria nella quale stai attualmente frequentando il corso di specializzazione? *

Contrassegna solo un ovale.

- Sì
- No

12. In quale Università italiana hai conseguito la Laurea in Medicina e Chirurgia? *

____

Passa alla domanda 13.

Comunicazione

Pensando al Tuo percorso di medico in formazione in Chirurgia Generale

13. Ti è capitato di dover *comunicare* una diagnosi *difficile* (es. diagnosi di cancro, "fine vita", errore medico/chirurgico) **da solo**, senza sostegno del tutor o di figure professionali con più esperienza di te a cui chiedere supporto? *

Contrassegna solo un ovale.

- Sì, spesso
- Sì, a volte
- Sì, raramente
- No, mai *Passa alla domanda 21.*

Passa alla domanda 14.

Esperienze comunicazione

Ripensando all'evento

14. Come ti sei sentita/o? *

Indica al massimo tre risposte tra le seguenti

Seleziona tutte le voci applicabili.

- [ ] Tranquilla/o
- [ ] Calma/o e sicura/o di me
- [ ] Irritata/o per essere da sola/o
- [ ] Spaventata/o
- [ ] Insicura/o (non sapevo spiegarmi bene)
- [ ] Imbarazzata/o (non avrei voluto trovarmi in quella situazione "senza strumenti")
- [ ] Mi sono vergognata/o di fronte a paziente e familiari

Come ritieni di aver *comunicato*?

15. Penso di essere stata/o chiara/o *

Contrassegna solo un ovale.

	1	2	3	4	5	
poco	◯	◯	◯	◯	◯	molto

16. Penso che il/la paziente mi abbia compresa/o *

Contrassegna solo un ovale.

	1	2	3	4	5	
poco	◯	◯	◯	◯	◯	molto

17. Penso di aver avuto un atteggiamento empatico circa emozioni e stati d'animo del/la paziente *

Contrassegna solo un ovale.

	1	2	3	4	5	
poco	◯	◯	◯	◯	◯	molto

18. Da 1 a 10 quanto ti ritieni soddisfatta/o della tua comunicazione? *

Contrassegna solo un ovale.

	1	2	3	4	5	6	7	8	9	10	
poco	◯	◯	◯	◯	◯	◯	◯	◯	◯	◯	molto soddifatto

19. Come avresti voluto affrontare quella comunicazione? *

Indica, tra le seguenti, l'**unica** risposta che ritieni più importante

Contrassegna solo un ovale.

- ◯ Con il supporto del tutor
- ◯ Sapendo come "impostare" la comunicazione
- ◯ Sapendo come gestire le "emozioni" dell'interlocutore
- ◯ Anche da sola/o ma con maggior sicurezza e tranquillità, avendo acquisito preliminarmente i "mezzi" per comunicare correttamente
- ◯ Credo di aver affrontato la comunicazione proprio come avrei desiderato affrontarla

20. Quali strumenti avevi (o ti erano stati forniti) per affrontare quella conversazione? *

Contrassegna solo un ovale.

- Non mi sono stati forniti strumenti specifici
- Ho assistito alle visite del mio Tutor e/o altri Professori/professionisti e mi sono fatta/o "un'idea" di come si comunicano le diagnosi "difficili" (in positivo e negativo)
- Mi sono documentata/o personalmente a riguardo (letteratura scientifica/libri/web/corsi/etc.)
- Durante il percorso di laurea ho ricevuto una formazione specifica sulla comunicazione medico-paziente e sulla comunicazione delle "bad-news"

Passa alla domanda 26.

Formazione

21. Nella Scuola di Specializzazione che stai attualmente frequentando, o che hai frequentato, sono previsti programmi, incontri formativi o corsi *specifici* su questi argomenti (*comunicazione medico-paziente, comunicazione diagnosi oncologiche, comunicazione bad-news*)? *

Contrassegna solo un ovale.

- Sì
- No
- Non ne sono a conoscenza

22. Ritieni che tra le aspettative di formazione di un medico in formazione in Chirurgia generale, sia importante tutelare anche questo aspetto, ovvero la formazione in ambito "*comunicativo-relazionale*" con il paziente, in particolare oncologico? *

Contrassegna solo un ovale.

	1	2	3	4	5	
poco	◯	◯	◯	◯	◯	molto

23. Qualora tu abbia risposto Sì alla domanda precedente, quanto ritieni importante questo aspetto in una scala da 1 a 10 nella formazione di uno Specialista in Chirurgia Generale? *

Contrassegna solo un ovale.

	1	2	3	4	5	6	7	8	9	10	
poco	◯	◯	◯	◯	◯	◯	◯	◯	◯	◯	molto importante

24. Ti piacerebbe (o ti sarebbe piaciuto) che la tua formazione comprendesse questo insegnamento, ovvero una "*educazione alla comunicazione di qualità in chirurgia oncologica*" ? *

Contrassegna solo un ovale.

	1	2	3	4	5	
poco	◯	◯	◯	◯	◯	molto

25. Come vorresti che venisse erogata questo tipo di formazione? *

Seleziona tutte le voci applicabili.

- [ ] lezioni frontali
- [ ] Didattica attiva (giochi di ruolo, simulazioni, esercitazioni pratiche, etc.)
- [ ] seminari online mensili/bimestrali
- [ ] coinvolgimento di professionisti con formazione specifica
- [ ] altro

Passa alla domanda 31.

Stress e Burn-out

Ripensando a come ti sei sentito dopo l'evento

26. Ti sei sentito coinvolta/o emotivamente dal tuo lavoro? *

Contrassegna solo un ovale.

	1	2	3	4	5	
poco	◯	◯	◯	◯	◯	molto

27. Ti sei immedesimata/o facilmente nei sentimenti del paziente?

Contrassegna solo un ovale.

	1	2	3	4	5	
poco	◯	◯	◯	◯	◯	molto

28. Ti sei sentita/o "consumata/o" dal tuo lavoro?

Contrassegna solo un ovale.

	1	2	3	4	5	
poco	◯	◯	◯	◯	◯	molto

29. Ti sei preoccupata/o che questo lavoro ti renda insensibile?

Contrassegna solo un ovale.

	1	2	3	4	5	
poco	○	○	○	○	○	molto

30. Ti sei sentita/o esaurita/o a fine giornata e ti è sembrato di essere più irritabile del dovuto?

Contrassegna solo un ovale.

	1	2	3	4	5	
poco	○	○	○	○	○	molto

Passa alla domanda 21.

Altri commenti liberi

Tutti i suggerimenti e i tuoi commenti a questa survey sono i benvenuti!

31. Puoi lasciare qui il tuo messaggio di testo facoltativo.

Questi contenuti non sono creati né avallati da Google.

Google Moduli
