# Supplementary material for: Communication skill training in surgical residency: insights from Y-SICO (Young-Italian Society of Surgical Oncology)
Source: Updates Surg. 2026 Apr 10;78(4):1811–24. doi: 10.1007/s13304-026-02557-2 (PMC13421255; doi:10.1007/s13304-026-02557-2)
Supplement: Supplementary file 2 — Supplementary file2 (PDF 212 KB) [file 13304_2026_2557_MOESM2_ESM.pdf]

Survey Young-SICO "COSTRUIRE": COmmunication Skills TRaining in sUrgIcal REsidency
How do we communicate a cancer diagnosis?

Dear Colleague,

We would like to thank you for your kind availability to collaborate in this research, which is promoted by Young SICO (Italian Society of Surgical Oncology).
The questionnaire we are sharing is brief (approx. 5 minutes to complete) and aims to capture and better understand the current state of communication training offered to general surgery residents and recently graduated surgeons (with a maximum of 3 years' work experience), particularly with regard to "medical communication" in oncologic settings.

There are no "right" or "wrong" answers to the questions below—feel free to select the responses that best reflect your experience and perspective.

We sincerely thank you in advance for your time and participation. By taking part in this survey, you will be included as a member of the "Collaborative Group" authorship of this study.

For this reason, we will ask to provide your name and surname at the beginning of the questionnaire. However, an identification number will then be assigned to you by the system to ensure your privacy. Your personal information will be kept strictly confidential.

See you soon!
Y-SICO Board

1. Informed Consent

Select one option only:
○ I agree
○ I do not agree

General Information

2. Identification

Full Name *
[Open text field]

3. How old are you?

[Open text field]

4. Professional status.

Are you currently a resident in General Surgery, or did you complete residency in the same field recently?

Select one option only:
○ I am a general surgery resident → Skip to question 9
○ I completed my general surgery residency less than 3 years ago → Continue to question 5
○ I completed my general surgery residency more than 3 years ago → Continue to question 5
○ I am a newly certified general surgeon

[If the participant is a current resident, they are directed to question 9]

Early-career general surgeon Pathway

5. Which General Surgery Residency Program did you attend?

[Open text field]

6. How many years ago did you complete your residency?

[Open text field]

7. Did you earn your MD degree at the same university where you completed your residency?

○ Yes
○ No

8. At which Italian university did you earn your MD degree?

[Open text field]

Resident Pathway

9. At which General Surgery School are you currently enrolled?

[Open text field]

10. What year of residency are you currently in?

○ First
○ Second
○ Third
○ Fourth
○ Fifth

11. Did you earn your MD degree at the same university where you are currently doing your residency?

○ Yes
○ No

12. At which Italian university did you earn your medical degree?

[Open text field]

Communication Experiences

13. Thinking about your training as a General Surgery resident, have you ever had to deliver a difficult diagnosis (e.g. cancer diagnosis, end-of-life communication, medical/surgical error) by yourself, without the support of a tutor or a more experienced professional?

○ Yes, often
○ Yes, sometimes
○ Yes, rarely
○ No, never → Skip to question 21

14. How did you feel in that situation? ***(Select up to three options)***

☐ Calm
☐ Confident and in control
☐ Frustrated for being alone
☐ Scared
☐ Insecure (I didn't know how to express myself)
☐ Embarrassed (I didn't want to be in that situation "without tools")
☐ Ashamed in front of the patient and family

15. How clearly do you think you communicated?

(Scale: 1 = not clear at all, 5 = very clear)
○ 1 ○ 2 ○ 3 ○ 4 ○ 5

16. Do you think the patient understood you?

(Scale: 1 = not at all, 5 = completely)
○ 1 ○ 2 ○ 3 ○ 4 ○ 5

17. How did you feel during the conversation?

(Scale: 1 = very uncomfortable, 5 = very comfortable)
○ 1 ○ 2 ○ 3 ○ 4 ○ 5

18. From 1 to 10, how satisfied are you with how you handled the communication?

○ 1 ○ 2 ○ 3 ○ 4 ○ 5 ○ 6 ○ 7 ○ 8 ○ 9 ○ 10

19. How would you have liked to handle that conversation? ***(Choose the most important aspect)***

○ With the support of a tutor
○ Knowing how to structure the conversation
○ Knowing how to manage the patient's emotions
○ By myself but with more confidence and the right tools
○ Exactly the way I handled it
○ By showing empathy toward the patient's feelings and emotional state

20. What tools did you have (or were given) to handle that conversation?

○ I was not given any specific tools
○ I observed my tutor or other professionals and got a sense of how to communicate "difficult" diagnoses (for better or worse)
○ I looked for information on my own (scientific literature, books, web, courses, etc.)
○ I received formal training on communication during medical school (doctor-patient interaction and breaking bad news)

Training

21. In the General Surgery Residency Program you are currently attending (or have completed), are there any specific courses, training sessions, or meetings on topics such as doctor–patient communication, cancer diagnosis disclosure, or breaking bad news?

○ Yes
○ No
○ I'm not aware of any

22. Do you think that communication training was sufficiently addressed during your residency?

(Scale: 1 = not at all, 5 = very much)
○ 1 ○ 2 ○ 3 ○ 4 ○ 5

23. If you answered "yes" to the previous question, how important do you think communication training is in the education of a General Surgery specialist?

(Scale: 1 = not important at all, 10 = extremely important)
○ 1 ○ 2 ○ 3 ○ 4 ○ 5 ○ 6 ○ 7 ○ 8 ○ 9 ○ 10

24. Would you have liked (or would you like) your training to include specific education in communication skills, especially related to cancer surgery?

○ Yes
○ No

25. How would you prefer this type of training to be delivered? ***(Select all that apply)***

☐ Lectures
☐ Interactive methods (role-playing, simulations, practical exercises, etc.)
☐ Monthly or bimonthly online seminars
☐ Involvement of professionals with specific training in communication
☐ Other: _______

Stress and Burnout

Thinking back to how you felt after the event…

26. Did you feel emotionally affected by your work?

(Scale: 1 = not at all, 5 = very much)
○ 1 ○ 2 ○ 3 ○ 4 ○ 5

27. Did you find it easy to empathize with the patient's feelings?

(Scale: 1 = not at all, 5 = very much)
○ 1 ○ 2 ○ 3 ○ 4 ○ 5

28. Did you feel "worn out" by your work?

(Scale: 1 = not at all, 5 = very much)
○ 1 ○ 2 ○ 3 ○ 4 ○ 5

29. Were you concerned that this kind of work might make you emotionally numb?

(Scale: 1 = not at all, 5 = very much)
○ 1 ○ 2 ○ 3 ○ 4 ○ 5

30. Did you feel exhausted at the end of the day, and more irritable than usual?

(Scale: 1 = not at all, 5 = very much)
○ 1 ○ 2 ○ 3 ○ 4 ○ 5

Other Comments

31. Any final thoughts or suggestions?

We welcome all your comments and feedback about this survey.
[Open text field]
