# Supplementary material for: Communication skill training in surgical residency: insights from Y-SICO (Young-Italian Society of Surgical Oncology)
Source: Updates Surg. 2026 Apr 10;78(4):1811–24. doi: 10.1007/s13304-026-02557-2 (PMC13421255; doi:10.1007/s13304-026-02557-2)
Supplement: Supplementary file 3 — Supplementary file3 (DOCX 18 KB) [file 13304_2026_2557_MOESM3_ESM.docx]

COSTRUIRE-Collaborative Group

Caterina Accardo, Elisa Agus, Luca Alberti, Maria Ida Amabile, Carla Ammendolia, Giorgio Ammerata, Paolo Amoretti, Felicia Andrei, Michela Angelucci, Alfredo Annicchiarico, Pietro Anoldo, Laura Antolino, Valerio Argiolas, Mirko Armas, Enrica Avezzù, Andrea Baldo, Lorenzo Barberis, Maria Rachele Barbieri, Giulia Becherucci, Elisa Bertilone, Chiara Bettini, Massimo Biondo, Francesca Blasa, Andrea Boniotti, Giacomo Borroni, Alessandra Bozza, Greta Bracchetti, Francesco Brucchi, Angela Bucaro, Anna Bella Burciu, Fabiana Caciolo, Riccardo Calef, Matteo Calì, Gaetano Silvio Calleri, Federico Cammillini, Vincenzo Canalella, Fabio Carbone, Barbara Carlucci, Andrea Castriotti, Livio Catozzi, Giulia Chiappini, Nicola Cillara, Matteo Cinquepalmi, Enrico Coletta, Gaia Colletti, Luigi Eduardo Conte, Sophia Costacurta, Antonio Costanzo, Luciano Curella, Carmen Cutolo, Fabrizio D'Acapito, Chiara D’Alterio, Giuliano D'Onghia, Federica De Franco, Renato De Martino, Paola De Nardi, Giovanni De Nobili, Giuseppe De Ruggieri, Matteo Desio, Agnese Dezi, Giuseppe Lorenzo Di Giulio, Giulia Di Lieto, Marcello Di Martino, Giovanna Di Meo, Giulia Di Raimondo, Tommaso Dominioni, Claudia Donello, Miriam Attalla El Halabieh, Tal Deborah Engel, Lorenzo Epis, Anna Esposito, Anna Falasca, Giacinto Falco, Agostino Fernicola, Francesco Ferrara, Davide Ferrari, Lorenzo Ferri, Enrico Fischetti, Gianluca Fiumara, Laura Fortuna, Antonio Franzese, Martina Fricano, Gaetano Gallo, Alessia Galvano, Tiziana Garritano, Patrizia Alba Gentile, Giulia Germiniasi, Marco Giacometti, Mauro Giambusso, Andrea Gioffré, Gennaro Giovine, Giuseppe Giuliani, Alice Gori, Lorenzo Gozzini, Giulia Grassi, Antonella Grasso, Serena Guarriello, Maryam Hosseinpour, Ilda Hoxhaj, Alessandro Iacomino, Roberta Iadarola, Maria Iannello, Eva Iannuzzi, Luca Improta, Luca Ippolito, Roberta La Mendola, Annarita Libia, Gabriella Lionetto, Antonio Luberto, Fabrizio Luca, Claudio Luciani, Michele Manara, Serena Mantova, Chiara Marafante, Paolo Maresca, Giancarlo Maresca, Martina Marrelli, Patrizia Marsanic, Matteo Mascherini, Alberto Massocco, Manuela Mastronardi, Marco Domenico Mazza, Gennaro Mazzarella, Gennaro Melone, Paolo Enrico Meneghesso, Valentina Messina, Valentina Miacci, Flavio Milana, Margherita Minghetti, Marica Mirabella, Rosario Minà, Perla Molica, Serena Molica, Federico Morabito, Marika Morabito, Andrea Morini, Rossella Moscatiello, Edoardo Mosciatti, Marco Nicolazzi, Stefania Nigro, Cecilia Orsini, Chiara Pagnoni, Giuseppe Palomba, Elisa Paoluzzi Tomada, Vincenzo Papagni, Roberto Passa, Carola Perinotti, Bruno Perotti, Teresa Perra, Giovanni Piazza, Sara Pollesel, Gianmario Edoardo Poto, Silvia Puddu, Emanuela Querci, Valeria Quintodei, Serena Ragonici, Mario Rampa, Emanuele Rausa, Luca Resca, Valerio Rinaldi, Luca Risi, Nicola Rocco, Fausto Rosa, Leonardo Rossi, Edoardo Saladino, Giacomo Salina, Anna Sanfilippo, Pietro Santocchi, Matteo Santoliquido, Paolina Saullo, Stefania Saverino, Valentina Sbacco, Lorenzo Scardina, Andrea Scardino, Alessia Scarton, Federica Scolari, Alessandra Scotto di Uccio, Giuseppe Sena, Rosina Siciliano, Leandro Siragusa, Alessandro Soave, Marco Summa, Flavia Taglioni, Giorgio Talamo, Francesco Taliente, Marsia Tancredi, Silvia Tedesco, Ilaria Tersigni, Flavio Tirelli, Antonio Toesca, Giovanni Tomasicchio, Beatrice Torre, Irene Tucceri Cimini, Alessio Vagliasindi, Marina Valente, Mariafelicia Valeriani, Angelo Maria Velardi, Alessandro Veltri, Luca Ventrone, Tommaso Violante, Mario Gaetano Visaloco, Davide Zattoni, Iris Zoto.
