# Supplementary material for: Communication skill training in surgical residency: insights from Y-SICO (Young-Italian Society of Surgical Oncology)
Source: Updates Surg. 2026 Apr 10;78(4):1811–24. doi: 10.1007/s13304-026-02557-2 (PMC13421255; doi:10.1007/s13304-026-02557-2)
Supplement: Supplementary file 4 — Supplementary file4 (DOCX 17 KB) [file 13304_2026_2557_MOESM4_ESM.docx]

| **List of Attended General Surgery Residency Programs** | **Number** |  | **List of Medical Schools Attended** | **Number** |
| --- | --- | --- | --- | --- |
| Università degli Studi Sapienza di Roma | 12 |  | Università degli Studi Sapienza di Roma | 15 |
| Università Cattolica del Sacro Cuore di Roma | 10 |  | Università Cattolica del Sacro Cuore di Roma | 7 |
| Università degli Studi di Verona | 5 |  | Università degli Studi di Napoli - Federico II | 7 |
| Università degli Studi di Milano | 5 |  | Università degli Studi di Messina | 4 |
| Università degli Studi di Padova | 4 |  | Università degli Studi di Roma Tor Vergata | 3 |
| Università degli Studi di Napoli - Federico II | 4 |  | Università degli Studi di Palermo | 3 |
| Università dell'Insubria - Varese | 3 |  | Università degli Studi di Milano | 2 |
| Università degli Studi di Parma | 3 |  | Università degli Studi di Genova | 2 |
| Università degli Studi di Siena | 3 |  | Università degli Studi di Parma | 2 |
| Università degli Studi di Bari | 2 |  | Università degli Studi di Bari | 2 |
| Università degli Studi Humanitas di Milano | 2 |  | Università dell'Insubria - Varese | 2 |
| Università degli Studi di Brescia | 1 |  | Università degli Studi di Verona | 2 |
| Università Campus Bio-Medico Di Roma | 3 |  | Università degli Studi diParma | 1 |
| Università degli Studi di Catanzaro | 1 |  | Università degli Studi dell'Aquila | 2 |
| Università degli Studi di Pisa | 1 |  | Università degli Studi di Catania | 1 |
| Università degli Studi "G. D'Annunzio" Chieti Pescara | 1 |  | Università degli Studi di Perugia | 1 |
| Università degli Studi di Foggia | 1 |  | Università degli Studi di Campus Bio-Medico di Roma | 1 |
| Università degli Studi di Tor Vergata di Roma | 1 |  | Università degli Studi di Catanzaro | 1 |
| Università degli Studi Di Salerno | 1 |  | Università degli Studi di "G. D'Annunzio" Chieti Pescara | 1 |
| Università degli Studi Di Sassari | 1 |  | Università degli Studi San Raffaele di Milano | 1 |
| Università degli Studi di Bologna | 1 |  | Università degli Studi di Trieste | 1 |
| Università degli Studi Di Genova | 2 |  | Università Degli Studi Di Cagliari | 1 |
| Università degli Studi di Madrid | 1 |  | Università degli Studi di Padova | 1 |
| Università degli Studi Di Messina | 1 |  | Università degli Studi di Pisa | 2 |
| Università degli Studi di Catanzaro | 1 |  | Università degli Studi di Salerno | 1 |
| Università degli Studi di Torino | 1 |  | Università degli Studi di Bologna | 1 |
| Università degli Studi di Modena e Reggio Emilia | 1 |  | Università degli Studi di Siena | 2 |
| Università degli Studi di Firenze | 1 |  | Università degli Studi di "Magna Graecia" di Catanzaro | 1 |
|  |  |  | Università degli Studi di Pavia | 1 |
|  |  |  | Università degli Studi di Torino | 1 |
|  |  |  | Università degli Studi di Modena e Reggio Emilia | 1 |
