# Supplementary material for: Communication skill training in surgical residency: insights from Y-SICO (Young-Italian Society of Surgical Oncology)
Source: Updates Surg. 2026 Apr 10;78(4):1811–24. doi: 10.1007/s13304-026-02557-2 (PMC13421255; doi:10.1007/s13304-026-02557-2)
Supplement: Supplementary file 5 — Supplementary file5 (DOCX 17 KB) [file 13304_2026_2557_MOESM5_ESM.docx]

| **List of General Surgery Residency Programs** | **Number** |  | **List of Medical Schools Attended** | **Number** |
| --- | --- | --- | --- | --- |
| Università degli Studi Sapienza di Roma | 18 |  | Università degli Studi di Roma Sapienza | 23 |
| Università degli Studi di Milano | 14 |  | Università degli Studi di Messina | 6 |
| Università degli Studi di Messina | 9 |  | Università degli Studi di Bologna | 6 |
| Università Cattolica del Sacro Cuore di Roma | 9 |  | Università degli Studi di Milano | 6 |
| Università degli Studi di Cagliari | 7 |  | Università degli Studi di Cagliari | 6 |
| Università degli Studi di Brescia | 7 |  | Università degli Studi di Brescia | 5 |
| Università Campus Bio-Medico di Roma | 6 |  | Università degli Studi di Roma Tor Vergata | 5 |
| Università degli Studi di Bari | 5 |  | Università degli Studi di Torino | 5 |
| Università degli Studi di Siena | 5 |  | Università degli Studi di Padova | 4 |
| Università degli Studi di Bologna | 4 |  | Università degli Studi di Bari | 4 |
| Università degli Studi di Napoli Federico II | 4 |  | Università degli Studi di Perugia | 4 |
| Università degli Studi Humanitas | 4 |  | Università degli Studi di Palermo | 6 |
| Università degli Studi di Torino | 4 |  | Università degli Studi Cattolica del Sacro Cuore di Roma | 3 |
| Università degli Studi di Salerno | 3 |  | Università degli Studi di Salerno | 3 |
| Università degli Studi dell'Aquila | 2 |  | Università degli Studi di Napoli Federico II | 3 |
| Università degli Studi di Verona | 2 |  | Università della Campania Luigi Vanvitelli | 3 |
| Università degli Studi di Padova | 2 |  | Università Politecnica delle Marche | 2 |
| Università degli Studi di Palermo | 2 |  | Università degli Studi dell'Aquila | 2 |
| Università Vita-Salute San Raffaele | 2 |  | Università degli Studi di Sassari | 2 |
| Università degli Studi di Roma Tor Vergata | 2 |  | Not Italy | 2 |
| Università degli Studi di Varese - Insubria | 1 |  | Università degli Studi di Catania | 2 |
| Università degli Studi "Magna Graecia" di Catanzaro | 1 |  | Università Magna Graecia di Catanzaro | 2 |
| Università degli Studi del Piemonte Orientale | 1 |  | Università degli Studi di Pisa | 1 |
| Università degli Studi di Milano-Bicocca | 1 |  | Università degli Studi G. d’Annunzio Chieti – Pescara | 1 |
| Università degli Studi di Trieste | 1 |  | Università degli Studi di Foggia | 1 |
| Università degli Studi di Perugia | 1 |  | Università degli Studi del Molise | 1 |
|  |  |  | Università degli Studi di Ferrara | 1 |
|  |  |  | Università del Piemonte Orientale | 1 |
|  |  |  | Università degli Studi di Genova | 1 |
|  |  |  | Università Campus Bio-Medico di Roma | 1 |
|  |  |  | Università degli Studi di Pavia | 1 |
|  |  |  | Università degli Studi di Siena | 1 |
|  |  |  | Humanitas University | 1 |
|  |  |  | Università degli Studi di Verona | 1 |
